# The irreducibility of collective obligations

**DOI:** 10.1007/s11098-018-01236-2

**Published:** 2019-01-12

**Authors:** Allard Tamminga, Frank Hindriks

**Affiliations:** 1 Faculty of Philosophy, University of Groningen, Oude Boteringestraat 52, 9712 GL Groningen, The Netherlands; 2 Department of Philosophy and Religious Studies, Utrecht University, Janskerkhof 13, 3512 BL Utrecht, The Netherlands

**Keywords:** Collective obligation, Collective responsibility, Collectivism, Individualism, Deontic logic, Game theory

## Abstract

Individualists claim that collective obligations are reducible to the individual obligations of the collective’s members. Collectivists deny this. We set out to discover who is right by way of a deontic logic of collective action that models collective actions, abilities, obligations, and their interrelations. On the basis of our formal analysis, we argue that when assessing the obligations of an individual agent, we need to distinguish individual obligations from member obligations. If a collective has a collective obligation to bring about a particular state of affairs, then it might be that no individual in the collective has an individual obligation to bring about that state of affairs. What follows from a collective obligation is that each member of the collective has a member obligation to help ensure that the collective fulfills its collective obligation. In conclusion, we argue that our formal analysis supports collectivism.

## Introduction

Most benefits and harms in the socio-economic realm are brought about by collectives rather than individuals. Individuals can be held morally responsible for their actions, but does it also make sense to hold a collective morally responsible for the things it does or fails to do? An affirmative answer immediately raises the question of whether collective moral responsibility is reducible to the individual moral responsibility of the collective’s members. Individualists say yes, collectivists say no. If the individualists are right, what kind of reduction proves them right?

Two kinds of reduction are relevant here: (1) *logical reduction* concerns whether statements about collectives are logically equivalent to conjunctions of statements about individuals, and (2) *ontological reduction* concerns whether collective entities are just sets of individuals. For argumentative purposes, we start from the assumption that ontological reductionism is true, that is, we assume that a group is a set of individuals and that a group action is an ordered set of individual actions.^1^ We argue that, even if ontological reductionism is true, logical reductionism is false.

In this paper, we focus on the notion of collective obligation. Obligations are forward-looking responsibilities. They are also a good place to start for studying the reducibility of backward-looking responsibilities, at least in so far as blameworthiness is concerned. Not fulfilling an obligation is a necessary condition for being individually blameworthy: if an individual agent is individually blameworthy, then she failed to fulfill an obligation.^2^ Similarly, not fulfilling a collective obligation is a necessary condition for being collectively blameworthy: if a group is collectively blameworthy, then it failed to fulfill a collective obligation. Because of this, we employ a two-step strategy to establish whether collective moral responsibility is logically reducible to individual moral responsibility. We first develop a formal theory of collective obligations and then use the formal properties we have identified to find out how to evaluate the actions of individual group members in case a group of agents does not fulfill its collective obligation.

The structure of our paper is as follows. In Sect. 2, we explain why, in studying collective moral responsibility, it is important to develop a logical theory of collective actions and obligations. In Sect. 3, we present our deontic logic of collective action. This deontic logic is then used to study (1) logical relations between collective actions and individual actions, and (2) logical relations between collective obligations and individual obligations. We list some intuitive principles concerning collective actions and obligations and use our deontic logic to determine whether or not they are valid. In Sect. 4, we submit that when assessing the obligations of an individual agent, we must distinguish *individual* obligations from *member* obligations. We argue that, although collective obligations are *not* logically reducible to individual obligations, given some well-defined assumptions they do imply member obligations. What follows from a collective obligation is that each individual has a member obligation to help ensure that the collective fulfills its collective obligation. In conclusion, we argue that, because member obligations presuppose collective obligations, our formal analysis supports collectivism.

## Logical puzzles

The study of collective moral responsibility would be facilitated by a formal theory that systematically distinguishes valid principles about collective actions and obligations from invalid ones. Unfortunately, a formal theory of this sort has yet to be offered. Most of the existing literature on collective moral responsibility remains informal and relies on everyday examples. Feinberg (1968) discusses the Jesse James train robbery and concludes that the passengers are collectively responsible for not having prevented it. Held (1970) takes us to a subway car in which five bystanders watch someone being strangled and also concludes that the collective is responsible.^3^ Sometimes such everyday examples are used as counterexamples to show that specific logical principles connecting collective actions and obligations with individual ones are *invalid*.^4^ So far so good.

But are there also *valid* principles that connect collective actions and obligations with individual ones? Frank Jackson thinks there are: ‘it is surely unbelievable that there are *no* valid principles linking the moral status of group acts with the moral status of constituent individual acts’ (Jackson 1987, p. 107). If we try to show that Jackson is right, we are presented with a difficulty: although everyday examples can be used to argue that some logical principles are *invalid*, they cannot be used to argue that some such principles are *valid*. To identify such validities, and also to systematize the scattered invalidities that were identified with the help of everyday examples, we need a formal theory of collective actions and obligations. This is by no means a straightforward matter.

Various authors appear to be puzzled about such a logic of collective actions and obligations. Let us consider two examples. (1) Kutz (2000a, p. 105) asserts that it is ‘puzzling’ that the conditional ‘If agent *i* and agent *j* together see to it that $\phi$, then agent *i* sees to it that $\phi$ and agent *j* sees to it that $\phi$’ is invalid, ‘while ordinarily each conjunct of a true conjunction is also true’. (2) Lawford-Smith (2012, pp. 455–456) maintains that the conditional ‘If agent *i* ought to see to it that $\phi \wedge \psi$, then agent *i* ought to see to it that $\phi$ and agent *i* ought to see to it that $\psi$’ and the conditional ‘If agent *i* and agent *j* together ought to see to it that $\phi$, then agent *i* ought to see to it that $\phi$ and agent *j* ought to see to it that $\phi$’ are both substitution instances of the logical principle of the distribution of ‘oughts’ over a conjunction. Because she proposes that we reject the second conditional, she questions the principle’s validity. The deontic logic of collective action that we present in Sect. 3 does of course vindicate the validity of the logical principle that the truth of a conjunction implies the truth of both of its conjuncts. Nonetheless, our deontic logic indicates why Kutz’s conditional is invalid, why Lawford-Smith’s first conditional is valid, and why her second conditional is not. That we can draw such conclusions illustrates the added value of our logic.

In Sects. 3 and 4 we develop a formal framework to study the logical relations between collective actions and obligations on the one hand and individual actions and obligations on the other. Our framework will remove puzzlement about the formal properties of collective action and obligation, and, more generally, provide a tool to investigate the formal relations between collective and individual responsibility. The framework will therefore also help us to achieve our second goal, which is to clarify the concept of logical reduction in the debate on individualism versus collectivism about collective moral responsibility. For these reasons, we construct a deontic logic of collective action.

## A deontic logic of collective action

The deontic logic of collective action presented in this section is inspired by John Horty’s (1996, 2001) deontic logic of agency.^5^ The present section and the next extend Horty’s ideas to a formal theory of collective obligations. Our theory provides clear answers to central questions about the relation between collective and individual obligations. Can it be the case that an individual in a collective ought to bring about a certain state of affairs without its being the case that that collective itself ought to bring about that state of affairs? (Yes.) Can it be the case that a collective ought to see to it that a certain state of affairs obtains and that an individual in that collective ought to see to it that that state of affairs does not obtain? (No.) Are the obligations of a group and all of its subgroups consistent? (Yes.)

To investigate formal relations between collective and individual obligations, we make two central idealizing assumptions to filter out factors that interfere with these relations to the point of trivializing them. Because there are no necessary relations between obligations generated by different moral codes or between obligations at different moments in time, we study, first, collective and individual obligations that are generated by the same moral code and, secondly, collective and individual obligations that hold at a single moment in time.

We use the term ‘group’ for any random collection of individuals (Held 1970). As a consequence, any combination of individual actions performed by such a random collection of individuals will be considered a group action performed by that random collective. Naturally, relaxing these assumptions may well result in different answers to the main questions about the relation between collective and individual obligations. It should therefore be borne in mind that our deontic logic of collective action is only a first step towards a fully-fledged formal analysis of collective actions and obligations.

Our deontic logic is, however, meant to be general in the following respect. Some group actions are just sets of individual actions. Think, for instance, of harming the ozone layer. Other group actions involve collective intentions, such as the collective intention to move the piano upstairs together. To attain a level of generality that enables us to study common features of group actions that involve collective intentions (like moving the piano upstairs) and of group actions that lack them (like harming the ozone layer), we initially assume that the sets of individuals who perform group actions have not formed a collective intention. On the basis of this assumption, we model group actions as sets of individual actions. Later on, we relax this assumption to accommodate joint actions generated by collective intentions as well.^6^ We model collective intentions in a minimal way as group plans that have been adopted by all members of the group.

This technical section proceeds as follows. First, we define our formal language and, by way of illustration, translate the statements that puzzled Kutz and Lawford-Smith into this language. Secondly, we define the deontic game models that interpret the formal language and then check whether the translations of Kutz’s and Lawford-Smith’s statements are valid, that is, whether the translations of these statements are true in all models. Thirdly, we justify the answers to the questions that opened this section by checking the validity of some general claims about relations between group actions and subgroup actions and between group obligations and subgroup obligations. All claims are proved in the "Appendix".

### Formal language

Our propositional modal language ${\mathfrak {L}}$ is built from a finite set $N=\{i^{}_1,\ldots ,i^{}_n\}$ of individual agents and a countable set ${\mathfrak {P}}=\{p^{}_1,p^{}_2,\ldots \}$ of atomic formulas. The formal language ${\mathfrak {L}}$ is the smallest set (in terms of set-theoretical inclusion) that satisfies conditions (i) through (iv):

(i) $${\mathfrak {P}}\subseteq {\mathfrak {L}}$$
(ii) If $\phi \in {\mathfrak {L}}$, then $\lnot \phi \in {\mathfrak {L}}$ and $\Diamond \phi \in {\mathfrak {L}}$
(iii) If $\phi \in {\mathfrak {L}}$ and $\psi \in {\mathfrak {L}}$, then $(\phi \wedge \psi )\in {\mathfrak {L}}$
(iv) If ${\mathcal {G}}\subseteq N$ and $\phi \in {\mathfrak {L}}$, then $[{\mathcal {G}}]\phi \in {\mathfrak {L}}$ and $({\mathcal {G}})\phi \in {\mathfrak {L}}$.

We omit parentheses, brackets, and braces if the omission does not give rise to ambiguities. The operators $\vee$, $\rightarrow$, and $\Box$ abbreviate the usual constructions.

This propositional modal language can be used to express *alethic statements* like ‘It is possible that $\phi$’ (formalized as $\Diamond \phi$), *agentive statements* like ‘Group ${\mathcal {G}}$ of agents sees to it that $\phi$’ (formalized as $[{\mathcal {G}}]\phi$), and *deontic statements* like ‘Group ${\mathcal {G}}$ of agents ought to see to it that $\phi$’ (formalized as $({\mathcal {G}})\phi$). Abilities can then be expressed by a combination of operators: ‘Group ${\mathcal {G}}$ of agents has the ability to see to it that $\phi$’ is formalized as $\Diamond [{\mathcal {G}}]\phi$.^7^

The statements that puzzled Kutz (2000a, p. 105) and Lawford-Smith (2012, pp. 455–456) are:

1. If agent *i* and agent *j* together see to it that $\phi$, then agent *i* sees to it that $\phi$ and agent *j* sees to it that $\phi$.
2. If agent *i* ought to see to it that both $\phi$ and $\psi$, then agent *i* ought to see to it that $\phi$ and agent *i* ought to see to it that $\psi$.
3. If agent *i* and agent *j* together ought to see to it that $\phi$, then agent *i* ought to see to it that $\phi$ and agent *j* ought to see to it that $\phi$.

They can now be translated as ($1^{\prime}$), ($2^{\prime}$), and ($3^{\prime}$):

($$1^{\prime}$$)
$$[i,j]\phi \rightarrow ([i]\phi \wedge [j]\phi )$$
($$2^{\prime}$$)
$$(i)(\phi \wedge \psi )\rightarrow ((i)\phi \wedge (i)\psi )$$
($$3^{\prime}$$)
$$(i,j)\phi \rightarrow ((i)\phi \wedge (j)\phi )$$.

To show that ($2^{\prime}$) is valid and that ($1^{\prime}$) and ($3^{\prime}$) are not, we need to know the conditions under which a formula of our modal language is true. These truth-conditions are specified by our deontic game models.

### Deontic game models

What ingredients do we need to specify truth-conditions for agentive and deontic statements? Intuitively, the truth of an agentive statement like ‘Group ${\mathcal {G}}$ of agents sees to it that *ϕ*’ requires that group ${\mathcal {G}}$ choose and perform a group action from among a range of group actions that are available to the group and that by performing this group action the group ensures that $\phi$ is true. Moreover, if we order the available group actions in terms of how well they promote a given moral code, then, intuitively, the truth of a deontic statement like ‘Group ${\mathcal {G}}$ of agents ought to see to it that $\phi$’ requires that every best group action that is available to group ${\mathcal {G}}$ ensures, if performed, that $\phi$ is true.

To convert these intuitive requirements for the truth of agentive and deontic statements into truth-conditions, we use a model theory based on the possible-worlds semantics for *standard deontic logic* (Hilpinen 1971, pp. 13–15). A possible-worlds model for standard deontic logic involves a non-empty set of possible worlds, a set of possible worlds that are deontically ideal with respect to a given moral code (this set of deontically ideal possible worlds is a subset of the set of possible worlds), and a valuation function that assigns to each atomic formula a set of possible worlds where that formula is true. In such a possible-worlds model, a deontic statement like ‘It ought to be that $\phi$’ is true if and only if $\phi$ is true in all deontically ideal possible worlds.

Our *deontic game models* are possible-worlds models for standard deontic logic where the role of possible worlds is played by possible *action profiles* (to be defined below). Hence, a deontic game model consists of a set of possible action profiles, a set of possible action profiles that are deontically ideal with respect to a given moral code (this set of deontically ideal possible action profiles is a subset of the set of possible action profiles), and a valuation function that assigns to each atomic formula a set of possible action profiles where that formula is true.

Formally, a deontic game model involves a finite set $N=\{i^{}_1,\ldots ,i^{}_n\}$ of individual agents. Each agent *i* in *N* is assigned a non-empty and finite set $A^{}_i$ of available individual actions.^8^ We use $a^{}_i$ and $a^{\prime}_i$ as variables for actions in the set $A^{}_i$ of actions that are available to agent *i*. The set *A* of possible action profiles is given by the Cartesian product $\times ^{}_{i\in N}A^{}_i$ of all the individual agents’ sets of actions. Note that *A* ($=\times ^{}_{i\in N}A^{}_i$) is non-empty, because all the $A^{}_i$’s are non-empty. We use *a* and $a^{\prime}$ as variables for action profiles in the set *A* of possible action profiles.^9^ The set of deontically ideal possible action profiles (which is a subset of the set *A* of possible action profiles) is defined by a deontic ideality function *d* that assigns to each possible action profile *a* in *A* a value *d*(*a*) that is either 1 (if *a* is deontically ideal) or 0 (if *a* is not deontically ideal).^10^ A valuation function *v* assigns to each atomic formula *p* in ${\mathfrak {P}}$ a (possibly empty) set *v*(*p*) of possible action profiles.

As an illustration, let us define a deontic game model $M_1$, in which there are two agents *i* and *j*. Let $A^{}_i=\{a^{}_i,a^{\prime}_i\}$ and $A^{}_j=\{a^{}_j,a^{\prime}_j\}$, that is, the actions that are available to agent *i* are $a^{}_i$ and $a^{\prime}_i$, and the actions that are available to agent *j* are $a^{}_j$ and $a^{\prime}_j$. The full set *A* of possible action profiles is then given by $A^{}_i\times A^{}_j=\{(a^{}_i,a^{}_j),(a^{}_i,a^{\prime}_j),(a^{\prime}_i,a^{}_j),(a^{\prime}_i,a^{\prime}_j)\}$. We define the deontic ideality function *d* as follows:

$$\begin{aligned} d(a^{}_i,a^{}_j)=\,& {} 1 \\ d(a^{}_i,a^{\prime}_{j})=\,& {} 0 \\ d(a^{\prime}_i,a^{}_j)=\,& {} 0 \\ d(a^{\prime}_i,a^{\prime}_j)=\,& {} 1. \end{aligned}$$

Accordingly, the action profiles $(a^{}_i,a^{}_j)$ and $(a^{\prime}_i,a^{\prime}_j)$ are deontically ideal, and the action profiles $(a^{}_i,a^{\prime}_j)$ and $(a^{\prime}_i,a^{}_j)$ are not. Lastly, we define the valuation function *v* (the valuations of the other atomic formulas are left unspecified):

$$\begin{aligned} v(p)=\,& {} \{(a^{}_i,a^{}_j)\} \\ v(q)=\,& {} \{(a^{}_i,a^{}_j), (a^{}_i,a^{\prime}_j), (a^{\prime}_i,a^{\prime}_j)\}. \end{aligned}$$

Accordingly, the atomic formula *p* is true at action profile $(a^{}_i,a^{}_j)$ and false at the other action profiles, and the atomic formula *q* is false at action profile $(a^{\prime}_i,a ^{}_j)$ and true at the other action profiles.

**Fig. 1:**
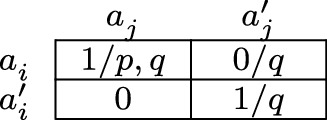
Deontic game model $M_1$

The deontic game model $M_1$ can be pictured as in Fig. 1. The rows represent the actions that are available to agent *i*, and the columns represent the actions that are available to agent *j*. Each cell therefore represents an action profile. Moreover, each cell contains a number that indicates whether or not the corresponding action profile is deontically ideal. Finally, we use the convention that a cell contains an atomic formula if and only if that atomic formula is true at the corresponding action profile.

We now turn to group actions. Following the literature on the logic of collective action,^11^ we think of an action performed by a group as a combination of individual actions performed by the group’s members. In the present setting, we make the idealizing assumption that, conversely, any combination of individual actions performed by the members of any group is a group action performed by that group (we relax this latter assumption in Sect. 4, where we introduce plan-based collective intentions which allow us to make a distinction between aggregate behaviour and group agency).

Accordingly, for each non-empty group ${\mathcal {G}}\subseteq N$ we define the set $A^{}_{\mathcal {G}}$ of group actions available to group ${\mathcal {G}}$ as $A^{}_{\mathcal {G}}=\times ^{}_{i\in {\mathcal {G}}}A^{}_i$. We use $a^{}_{\mathcal {G}}$ and $a^{\prime}_{\mathcal {G}}$ as variables for group actions in the set $A^{}_{\mathcal {G}}$ of group actions that are available to group ${\mathcal {G}}$. Given an action profile *a* in a deontic game model and a group ${\mathcal {G}}\subseteq N$, we also use $a^{}_{\mathcal {G}}$ to refer to the combination of individual actions of ${\mathcal {G}}$’s members in *a*. Likewise, we use $a^{}_{-{\mathcal {G}}}$ to refer to the combination of individual actions of all of ${\mathcal {G}}$’s non-members in *a*. For example, given an action profile $(a^{}_i,a^{}_j,a^{}_k,a^{}_l)$ in a four-agent deontic game model and the group ${\mathcal {G}}=\{i,k\}$, we have $a^{}_{\mathcal {G}}=(a^{}_i,a^{}_k)$ and $a^{}_{-{\mathcal {G}}}=(a^{}_j,a^{}_l)$. In the same fashion, we use $A^{}_{-{\mathcal {G}}}$ to refer to $\times ^{}_{i\in (N-{\mathcal {G}})}A^{}_i$.

Action profiles are fully determined by combinations of actions of all the individual agents. Hence, an agent only partly determines an action profile by performing an action, and this holds irrespective of whether the agent is an individual or a group. By performing an action an agent only makes a contribution to realizing an action profile. Nonetheless, by performing an action an agent can make statements true or false. In the present setting, given a specific action profile, a group sees to it that a particular statement is true if and only if the group’s contribution to this action profile suffices to ensure the truth of this statement, regardless of the actions of the group’s non-members. To be precise, given an action profile *a*, a group ${\mathcal {G}}$ sees to it that $\phi$ if and only if for all action profiles $a^{\prime}$ with $a^{\prime}_{\mathcal {G}}=a^{}_{\mathcal {G}}$ it holds that $\phi$ is true in $a^{\prime}$. (The truth-condition for individual agency is obtained by filling in $\{i\}$ for ${\mathcal {G}}$.)

We model group obligations as follows: a group ought to see to it that a particular statement is true if and only if all of its optimal actions ensure the truth of that statement. To discover which of the actions available to a group are optimal, we order the available actions in terms of the deontic ideality of their possible action profiles. We thus transform the given binary deontic ideality ordering of the action profiles in *A* into an ordering of the actions available to group ${\mathcal {G}}$, that is, into an ordering of $A^{}_{\mathcal {G}}$. The actions that are best in this ordering of $A^{}_{\mathcal {G}}$ are group ${\mathcal {G}}$’s optimal actions. Following Horty (2001, p. 68), we order a group’s available actions in terms of *dominance*. Given two actions $a^{}_{\mathcal {G}}$ and $a^{\prime}_{\mathcal {G}}$ that are available to a group ${\mathcal {G}}$, $a^{}_{\mathcal {G}}$ weakly dominates $a^{\prime}_{\mathcal {G}}$ (notation: $a^{}_{\mathcal {G}}\succeq a^{\prime}_{\mathcal {G}}$) if and only if $a^{}_{\mathcal {G}}$ promotes deontic ideality at least as well as $a^{\prime}_{\mathcal {G}}$, regardless of what the group’s non-members do:

#### **Definition 1**

(*Dominance*) Let $M=\langle N, (A^{}_i), d, v\rangle$ be a deontic game model. Let ${\mathcal {G}}\subseteq N$ be a group of agents. Let $a^{}_{\mathcal {G}},a^{\prime}_{\mathcal {G}}\in A^{}_{\mathcal {G}}$ be actions available to group ${\mathcal {G}}$. Then

$$\begin{aligned} a^{}_{\mathcal {G}}\succeq a^{\prime}_{\mathcal {G}}\,\,{\hbox {iff}}\,\,{\hbox {for}}\,\,{\hbox {all}}\,\,a^{\prime\prime}_{-{\mathcal {G}}}\in A^{}_{-{\mathcal {G}}}\,\,{\hbox {it}}\,\,{\hbox {holds}}\,\,{\hbox {that}}\,\,d\left( a^{}_{\mathcal {G}},a^{\prime\prime}_{-{\mathcal {G}}}\right) \ge d\left( a^{\prime}_{\mathcal {G}},a^{\prime\prime}_{-{\mathcal {G}}}\right) . \end{aligned}$$

Strong dominance is defined in terms of weak dominance: $a^{}_{\mathcal {G}}\succ a^{\prime}_{\mathcal {G}}$ if and only if $a^{}_{\mathcal {G}}\succeq a^{\prime}_{\mathcal {G}}$ and $a^{\prime}_{\mathcal {G}}\not \succeq a^{}_{\mathcal {G}}$.

A group’s optimal actions are the ones that are not strongly dominated:

#### **Definition 2**

(*Optimality*) Let $M=\langle N, (A^{}_i), d, v\rangle$ be a deontic game model. Let ${\mathcal {G}}\subseteq N$ be a group of agents. Then the set of ${\mathcal {G}}$’s *optimal actions*, denoted by $\textit{Optimal}({\mathcal {G}})$, is given by

$$\begin{aligned} \textit{Optimal}({\mathcal {G}}) = \left\{ a^{}_{\mathcal {G}}\in A^{}_{\mathcal {G}}:\,\,{\hbox {there}}\,\,{\hbox {is}}\,\,{\hbox {no}}\,\,a^{\prime}_{\mathcal {G}}\in A^{}_{\mathcal {G}}\,\,{\hbox {such}}\,\,{\hbox {that}}\,\,a^{\prime}_{\mathcal {G}}\succ a^{}_{\mathcal {G}}\right\} . \end{aligned}$$

Finally, we need to specify truth-conditions for the formulas of our propositional modal language. We write $M,a\models \phi$ if a formula $\phi$ is true at an action profile *a* of a deontic game model *M*. The truth-conditions are given inductively:^12^

#### **Definition 3**

(*Truth-conditions*) Let $M=\langle N, (A^{}_i), d, v\rangle$ be a deontic game model. Let ${\mathcal {G}}\subseteq N$ be a group of agents. Let $a\in A$ be an action profile. Let $p\in {\mathfrak {P}}$ be an atomic formula and $\phi ,\psi \in {\mathfrak {L}}$ arbitrary formulas. Then

$$\begin{aligned} \begin{array}{lll} M,a\models p &{} \hbox {iff} &{} a\in v(p) \\ M,a\models \lnot \phi &{} \hbox {iff} &{} M,a\not \models \phi \\ M,a\models \phi \wedge \psi &{} \hbox {iff} &{} M,a\models \phi \quad \hbox {and} \quad M,a\models \psi \\ M,a\models \Diamond \phi &{} \hbox {iff} &{} \hbox {there is an }a^{\prime}\in A\quad\hbox {such that}\quad M,a^{\prime}\models \phi \\ M,a\models [{\mathcal {G}}]\phi &{} \hbox {iff} &{} \hbox {for all } a^{\prime}\in A \quad\hbox {with}\quad a^{\prime}_{\mathcal {G}}=a^{}_{\mathcal {G}}\quad\hbox {it holds that}\quad M,a^{\prime}\models \phi \\ M,a\models ({\mathcal {G}})\phi &{} \hbox {iff} &{} \hbox {for all }a^{\prime}\in A \quad\hbox {with}\quad a^{\prime}_{\mathcal {G}}\in \textit{Optimal}({\mathcal {G}})\quad\hbox {it holds that}\quad M,a^{\prime}\models \phi . \end{array} \end{aligned}$$

We adopt the following standard conventions: given a deontic game model *M*, we write $M\models \phi$ if for all action profiles *a* in *A* it holds that $M,a\models \phi$. A formula $\phi$ is *valid* (notation: $\models \phi$) if for all deontic game models *M* it holds that $M\models \phi$.

### Validities and invalidities

We are now in a position to justify the answers to the opening questions of this technical section. To do so, we check the validity of some general claims about relations between group actions and obligations on the one hand and subgroup actions and obligations on the other. But first we make a general observation. Because deontic game models are possible-worlds models in disguise, our agentive and deontic group modalities inherit some basic properties of modalities whose truth-conditions are specified by universal conditions on possible worlds. Accordingly, all of our modalities are *normal* modal operators: they validate the principle of necessitation and the principle of distribution over implication.^13^ Although it is straightforward to define non-normal agentive and deontic group modalities on the basis of our normal ones, we avoid unnecessary complications and stick to normal group modalities in the theorems below.^14^ All claims are proved in the "Appendix".

Our first theorem states some of the basic properties of our deontic logic of collective action. First, if group ${\mathcal {G}}$ has an obligation to see to it that $\phi$, then group ${\mathcal {G}}$ has the ability to see to it that $\phi$, that is, it is possible that group ${\mathcal {G}}$ sees to it that $\phi$. This is the well-known principle that ‘ought’ implies ‘can’.^15^

Secondly, group ${\mathcal {G}}$ sees to it that both $\phi$ and $\psi$ if and only if both group ${\mathcal {G}}$ sees to it that $\phi$ and group ${\mathcal {G}}$ sees to it that $\psi$. Thirdly, group ${\mathcal {G}}$ ought to see to it that both $\phi$ and $\psi$ if and only if both group ${\mathcal {G}}$ ought to see to it that $\phi$ and group ${\mathcal {G}}$ ought to see to it that $\psi$. Note that the validity of the third claim implies that the statement ($2^{\prime}$) above, $(i)(\phi \wedge \psi )\rightarrow ((i)\phi \wedge (i)\psi )$, is also valid.

#### **Theorem 1**

*Let*$\phi ,\psi \in {\mathfrak {L}}$*and*${\mathcal {G}}\subseteq N$. *Then*

(i) $$\models ({\mathcal {G}})\phi \rightarrow \Diamond [{\mathcal {G}}]\phi $$
(ii) $$\models [{\mathcal {G}}](\phi \wedge \psi )\leftrightarrow ([{\mathcal {G}}]\phi \wedge [{\mathcal {G}}]\psi )$$
(iii) $$\models ({\mathcal {G}})(\phi \wedge \psi )\leftrightarrow (({\mathcal {G}})\phi \wedge ({\mathcal {G}})\psi )$$.

Our second theorem is on relations between group actions and subgroup actions. Let ${\mathcal {F}}$ be a subgroup of a group ${\mathcal {G}}$. First, if subgroup ${\mathcal {F}}$ sees to it that $\phi$, then group ${\mathcal {G}}$ sees to it that $\phi$.^16^ Secondly, the converse implication does not hold: it might be that group ${\mathcal {G}}$ sees to it that $\phi$, whereas subgroup ${\mathcal {F}}$ does not see to it that $\phi$. For a counterexample, note that in the deontic game model $M_1$ of Fig. 1, it holds that $M_1,(a^{}_i,a^{}_j)\models [i,j]p$ and $M_1,(a^{}_i,a^{}_j)\not \models [i]p$. Therefore, the statement ($1^{\prime}$) above, $[i,j]\phi \rightarrow ([i]\phi \wedge [j]\phi )$, is invalid.

#### **Theorem 2**

*Let*$$\phi \in {\mathfrak {L}}$$. *Then*

(i) $$\models [{\mathcal {F}}]\phi \rightarrow [{\mathcal {G}}]\phi $$ for all $${\mathcal {F}}\subseteq {\mathcal {G}}\subseteq N$$
(ii) $\not \models [{\mathcal {G}}]\phi \rightarrow [{\mathcal {F}}]\phi$ for some ${\mathcal {F}}\subseteq {\mathcal {G}}\subseteq N$.

Our third theorem is on relations between group obligations and subgroup obligations. Let ${\mathcal {F}}$ be a subgroup of a group ${\mathcal {G}}$. First, the fact that subgroup ${\mathcal {F}}$ ought to see to it that $\phi$ does not imply that group ${\mathcal {G}}$ ought to see to it that $\phi$: it might be that subgroup ${\mathcal {F}}$ has an obligation to see to it that $\phi$, whereas group ${\mathcal {G}}$ does not have an obligation to see to it that $\phi$. Secondly, the converse implication does not hold either: it might be that group ${\mathcal {G}}$ has an obligation to see to it that $\phi$, whereas subgroup ${\mathcal {F}}$ does not have an obligation to see to it that $\phi$. For a counterexample to the second claim, note that in the deontic game model $M_1$ of Fig. 1, for all action profiles *a* it holds that $M_1,a\models (i,j)q$ and $M_1,a\not \models (i)q$. Therefore, the statement ($3^{\prime}$) above, $(i,j)\phi \rightarrow ((i)\phi \wedge (j)\phi )$, is invalid.^17^

#### **Theorem 3**

*Let*$$\phi \in {\mathfrak {L}}$$. *Then*

(i) $$\not \models ({\mathcal {F}})\phi \rightarrow ({\mathcal {G}})\phi $$ for some $${\mathcal {F}}\subseteq {\mathcal {G}}\subseteq N$$
(ii) $\not \models ({\mathcal {G}})\phi \rightarrow ({\mathcal {F}})\phi$ for some ${\mathcal {F}}\subseteq {\mathcal {G}}\subseteq N$.

Our fourth theorem is also on relations between group obligations and subgroup obligations. Let ${\mathcal {F}}$ be a subgroup of group ${\mathcal {G}}$. First, if subgroup ${\mathcal {F}}$ ought to see to it that $\phi$ is true, then it is not the case that group ${\mathcal {G}}$ ought to see to it that $\phi$ is false. Secondly, if group ${\mathcal {G}}$ ought to see to it that $\phi$ is true, then it is not the case that subgroup ${\mathcal {F}}$ ought to see to it that $\phi$ is false.

#### **Theorem 4**

*Let*$\phi \in {\mathfrak {L}}$*and*${\mathcal {F}}\subseteq {\mathcal {G}}\subseteq N$. *Then*

(i) $$\models ({\mathcal {F}})\phi \rightarrow \lnot ({\mathcal {G}})\lnot \phi $$
(ii) $$\models ({\mathcal {G}})\phi \rightarrow \lnot ({\mathcal {F}})\lnot \phi $$.

Our fifth theorem is also on relations between group obligations and subgroup obligations, but now we consider two subgroups rather than one. To state the theorem clearly, we introduce some additional terminology. A group *has an obligation regarding*$\phi$ if it ought to see to it that $\phi$ is true or it ought to see to it that $\phi$ is false. Two groups *have contradictory obligations regarding*$\phi$, if the one ought to see to it that $\phi$ is true, whereas the other ought to see to it that $\phi$ is false. Two groups *have consistent obligations regarding*$\phi$, if they do not have contradictory obligations regarding $\phi$.

Let ${\mathcal {F}}^{}_1$ and ${\mathcal {F}}^{}_2$ be subgroups of a group ${\mathcal {G}}$. First, if two subgroups ${\mathcal {F}}^{}_1$ and ${\mathcal {F}}^{}_2$ have no common members, then their obligations regarding $\phi$ are consistent. Secondly, if two subgroups ${\mathcal {F}}^{}_1$ and ${\mathcal {F}}^{}_2$ do have common members, then they might have contradictory obligations regarding $\phi$. Thirdly, if two subgroups ${\mathcal {F}}^{}_1$ and ${\mathcal {F}}^{}_2$ of a group ${\mathcal {G}}$ have contradictory obligations regarding $\phi$, then group ${\mathcal {G}}$ does not have an obligation regarding $\phi$. Fourthly, and conversely, if group ${\mathcal {G}}$ has an obligation regarding $\phi$, then the obligations of its subgroups ${\mathcal {F}}^{}_1$ and ${\mathcal {F}}^{}_2$ regarding $\phi$ are consistent.

#### **Theorem 5**

*Let*$$\phi \in {\mathfrak {L}}$$. *Then*

(i) $\models ({\mathcal {F}}^{}_1)\phi \rightarrow \lnot ({\mathcal {F}}^{}_2)\lnot \phi$    for all ${\mathcal {F}}^{}_1,{\mathcal {F}}^{}_2\subseteq N$ such that ${\mathcal {F}}^{}_1\cap {\mathcal {F}}^{}_2=\emptyset$
(ii) $\not \models ({\mathcal {F}}^{}_1)\phi \rightarrow \lnot ({\mathcal {F}}^{}_2)\lnot \phi$    for some ${\mathcal {F}}^{}_1,{\mathcal {F}}^{}_2\subseteq N$
(iii) $\models (({\mathcal {F}}^{}_1)\phi \wedge ({\mathcal {F}}^{}_2)\lnot \phi )\rightarrow (\lnot ({\mathcal {G}})\phi \wedge \lnot ({\mathcal {G}})\lnot \phi )$    for all ${\mathcal {F}}^{}_1,{\mathcal {F}}^{}_2\subseteq {\mathcal {G}}\subseteq N$
(iv) $\models (({\mathcal {G}})\phi \vee ({\mathcal {G}})\lnot \phi )\rightarrow \lnot (({\mathcal {F}}^{}_1)\phi \wedge ({\mathcal {F}}^{}_2)\lnot \phi )$      for all ${\mathcal {F}}^{}_1,{\mathcal {F}}^{}_2\subseteq {\mathcal {G}}\subseteq N$.

To illustrate Theorem 5(ii), we need some seaside drama: far off the coast, a person is on the verge of drowning. He can be saved by Angela, Bob, and Charlotte, but only if they act together in specific pairs. Angela is an excellent swimmer, Bob a moderate one, and although Charlotte is a weak swimmer, she is the only one who can operate the motorized dinghy anchored nearby. If Angela and Bob work together they will be able to save the drowning person by swimming up to him immediately: Angela will arrive first, Bob somewhat later, and together they will bring the person back to shore. By contrast, if Bob and Charlotte work together they will be able to save the drowning person by using the dinghy: on arrival, Bob will jump into the water, and together he and Charlotte will manage to pull the person into the dinghy and bring him back to shore. Angela and Bob have a collective obligation to swim. Bob and Charlotte have a collective obligation not to swim. These two collective obligations cannot both be fulfilled. After all, the former requires both Angela and Bob to swim and the latter requires both Bob and Charlotte not to swim. This shows that subgroups that have common members can indeed have contradictory obligations regarding some proposition. Note also that the group consisting of Angela, Bob, and Charlotte has neither a collective obligation to swim nor a collective obligation not to swim.^18^

In sum, our deontic game models can be used to specify truth-conditions for formulas expressing group actions, abilities, obligations, and their interrelations. They thereby make it possible to check the validity of some central claims about relations between group obligations and subgroup obligations. In the next section, we argue that our deontic game models can also be used to make a principled distinction—crucial to our defence of collectivism about collective moral responsibility—between individual obligations and member obligations.

## Individual obligations and member obligations

If a group has a collective obligation and an individual wishes to assess her obligations, it matters whether the individual is a member of the group or not. To make sense of this simple observation within our formal framework, we make a principled distinction between individual obligations and member obligations and what it means to fulfill such obligations.^19^ An *individual obligation* is an obligation that an individual should fulfill regardless of the collective obligations of the groups of which she is a member. We say that an individual agent *fulfills* her individual obligation if and only if she performs one of her optimal individual actions. Likewise, a group fulfills its collective obligation if and only if it performs one of its optimal group actions. (Note that, just like an individual agent, a group of agents, by performing an optimal action, brings about all the states of affairs that it ought to bring about.) If an individual is a member of a group, a *member obligation* towards that group is an obligation that an individual should fulfill in order to help ensure that the group fulfills its collective obligation.^20^

How are we to understand the relation between collective obligations and member obligations? To answer this question, consider a strategic situation represented by a deontic game model. Assume that the individual agents involved are able to communicate and team up. Suppose that some individual agents decide to bundle their strengths and act as a team so as to be better able to promote deontic ideality.^21^ The members of the newly formed team will then have to determine which of their options are best, that is, which of the group actions available to them as a team are optimal. To discover these optimal group actions, the members of the team must reason from the standpoint of the team rather than from their individual perspectives. In doing so, they are engaged in *we-reasoning* (Hakli et al. 2010; Tuomela 2013).^22^ We take it that the outcome of this reasoning process is a set of optimal group actions. The team fulfills its collective obligation if and only if it performs one of those optimal group actions. Accordingly, the team members will have to design and adopt a *group plan* to coordinate their individual actions such that the composition of their individual actions amounts to an optimal group action (Tamminga and Duijf 2017, Section 3). The group plan thus specifies which individual actions the team members should perform to ensure that the team itself fulfills its collective obligation. Once the team members, aiming to fulfill a collective obligation, have adopted a group plan, a team member fulfills her *member obligation* towards the team if and only if she acts according to plan.

Transposing Bratman’s characterization of practical reasoning from an individualistic to a collective perspective, we can think of a group plan as providing ‘a filter on options that are potential solutions’ (Bratman 1987, p. 35) to a group’s coordination problem. Once adopted, a group plan coordinates the actions of the group members: it permits them to act *simultaneously* and *unconditionally*, in the full belief that every group member is acting according to plan. We make the natural assumption that after the group members have adopted a group plan to coordinate their actions, it is common knowledge among them that the plan has been adopted. The formation and adoption of a group plan therefore amounts to forming a collective intention. Group actions regulated by such a plan-based collective intention are known as *shared* or *joint actions*.

If there is a morally significant outcome that requires contributions from several individual agents, then each has an obligation to perform a relevant contributory action. In light of this we assume that when the group members adopt a group plan aimed at realizing a morally significant outcome, the individual members have an obligation to contribute to it.

Given a deontic game model in which a group of agents aims to fulfill its collective obligation, it will be convenient to think of a group plan as a non-empty subset of the set of the group’s optimal actions. By adopting a group plan, the group members agree to perform one of the group actions in the plan. To give a definition of how such a plan specifies the member obligations of the group members, let us first consider plans that consist of a single optimal group action (this might be just one out of several of the group’s optimal actions). Given a plan that consists of a single optimal group action, it is obvious that if each group member performs her contribution to that single optimal group action, then the group itself performs an optimal group action and thus fulfills its group obligation. We therefore propose the following definition: *given a plan consisting of a single optimal group action, a group member *fulfills* her member obligation specified by the plan if and only if she performs the action that is her component action of the single optimal group action in that plan.* (Things become more complicated, but also more realistic, if a plan consists of several distinct optimal group actions out of which an arbitrary one is to be performed—more on this at the end of this section.)

**Fig. 2:**
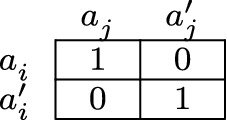
Not sufficient

**Fig. 3:**
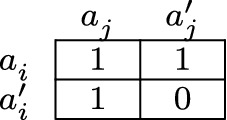
Not necessary

Individual obligations should be distinguished clearly from member obligations, because fulfilling an individual obligation is neither *necessary* nor *sufficient* for fulfilling a member obligation. This can be illustrated with the Figs. 2 and 3.^23^ To see that fulfilling an individual obligation is *not sufficient* for fulfilling a member obligation, consider the model of Fig. 2. There, it holds that agent *i* fulfills her individual obligation if and only if she performs one of the actions $a^{}_i$ and $a^{\prime}_i$, because both actions are optimal for her. Moreover, given the group plan $\{(a^{}_i,a^{}_j)\}$, agent *i* fulfills her member obligation specified by that plan if and only if she performs action $a^{}_i$. Hence, by performing action $a^{\prime}_i$ agent *i* fulfills her individual obligation without fulfilling her member obligation. Fulfilling an individual obligation is therefore *not sufficient* for fulfilling a member obligation.

To illustrate this, we return to the seaside, where a person is on the verge of drowning. Suppose now that Angela and Bob will save the drowning person if they both swim. They will also save the drowning person if they both use the motorized dinghy. If one of them swims and the other uses the boat, the person will drown. For Angela, it holds that if she has to act regardless of the collective obligations of the groups of which she is a member, then the two actions that are available to her—swimming and using the boat—are equally good: given that she must act alone, she does the best she can by performing either of these actions. Accordingly, were she to perform either of these two individually optimal actions, she would fulfill her individual obligation. Given that Angela and Bob coordinate their actions by adopting the group plan to save the drowning person by swimming, it holds that if Angela were to use the boat, then she would fulfill her individual obligation without fulfilling her member obligation.

To see that fulfilling an individual obligation is *not necessary* for fulfilling a member obligation, consider the model of Fig. 3. There, it holds that agent *i* fulfills her individual obligation if and only if she performs action $a^{}_i$, because it is her only optimal action. Moreover, given the group plan $\{(a^{\prime}_i,a^{}_j)\}$, agent *i* fulfills her member obligation specified by the plan if and only if she performs action $a^{\prime}_i$. Hence, by performing action $a^{\prime}_i$ agent *i* fulfills her member obligation without fulfilling her individual obligation. Fulfilling an individual obligation is therefore *not necessary* for fulfilling a member obligation. As a consequence, reasons for fulfilling an individual obligation might outweigh reasons for fulfilling a member obligation and vice versa.

To illustrate this, let us slightly change the seaside example. Suppose now that Angela and Bob can each save the drowning person on their own. Angela can perform either of two actions: swimming or not swimming. By swimming she will save the drowning person. Likewise, Bob can perform either of two actions: using the boat or not using the boat. By using the boat he will save the drowning person. For Angela, it holds that if she has to act regardless of the collective obligations of the groups of which she is a member, then the best she can do is to swim. Accordingly, she would fulfill her individual obligation were she to swim. Given that Angela and Bob coordinate their actions by adopting the group plan to save the drowning person by Angela’s not swimming and Bob’s using the boat, then it holds that if Angela were not to swim, she would fulfill her member obligation without fulfilling her individual obligation.

So far we have only studied member obligations specified by a plan consisting of a *single* optimal group action. This is unnecessarily restrictive and unrealistic. To allow for member obligations specified by plans that consist of several distinct optimal group actions, we propose the following general definition: *given a plan consisting of one or more optimal group actions, a group member *fulfills* her member obligation specified by the plan if and only if she performs an action that is her component action of one of the optimal group actions in that plan.*

It is important to note that a plan consisting of several distinct optimal group actions does not necessarily guarantee the truth of the conditional ‘If each group member fulfills her member obligation specified by the plan, then the group itself fulfills its group obligation’. To see this, let us take a second look at the model of Fig. 2. Consider the plan $\{(a^{}_i,a^{}_j),(a^{\prime}_i,a^{\prime}_j)\}$. Suppose that agent *i* performs action $a^{}_i$ and that agent *j* performs action $a^{\prime}_j$. Then both agent *i* and agent *j* fulfill their member obligation specified by the plan, although by performing the group action $(a^{}_i,a^{\prime}_j)$ the group does not fulfill its group obligation. This plan therefore fails to guarantee the truth of the conditional. It fails to solve the group members’ coordination problem. It would therefore be irrational to adopt such a plan. It is a *bad* plan.

A *good* plan—a rational plan—solves the group members’ coordination problem, that is, it guarantees the truth of the conditional ‘If each group member fulfills her member obligation specified by the plan, then the group itself fulfills its group obligation’.^24^ Equivalently, it guarantees the truth of the conditional ‘If the group does not fulfill its group obligation, then there is at least one group member who does not fulfill her member obligation specified by the plan’.^25^ A bad plan fails to guarantee the truth of these conditionals.

To ensure that a plan solves the group members’ coordination problem, the plan has to have a certain structure, namely that whichever group member performs whatever action that is her component action of one of the optimal group actions in the plan, the result is always one of the optimal group actions in the plan. To be precise, a plan should be *closed* under component individual actions:^26^

### **Definition 4**

(*Closedness*) Let $M=\langle N,(A^{}_i),d,v\rangle$ be a deontic game model. Let ${\mathcal {G}}\subseteq N$ be a group of agents. Let $B\subseteq A^{}_{\mathcal {G}}$. Then *B* is *closed* if and only if for all $a^{}_{\mathcal {G}},a^{\prime}_{\mathcal {G}}\in B$ and all $i\in {\mathcal {G}}$ it holds that $(a^{}_i,a^{\prime}_{{\mathcal {G}}-i})\in B$.

A few examples: $\{(a^{}_i,a^{\prime}_j)\}$ is closed (as is every set consisting of a single group action). Likewise, $\{(a^{}_i,a^{}_j),(a^{}_i,a^{\prime}_j)\}$ is closed. The set $\{(a^{}_i,a^{}_j),(a^{\prime}_i,a^{\prime}_j)\}$ is not, because $(a^{}_i,a^{\prime}_j)$ is not in it. The set $\{(a^{}_i,a^{}_j),(a^{}_i,a^{\prime}_j),(a^{\prime}_i,a^{}_j)\}$ is not closed, because $(a^{\prime}_i,a^{\prime}_j)$ is not in it.

A closed plan guarantees the truth of the conditional ‘If each group member fulfills her member obligation specified by the plan, then the group itself fulfills its group obligation’. If each group member fulfills her member obligation specified by a closed plan, each group member performs an action that is her component action of one of the optimal group actions in that plan. Because the plan is closed, it must be that this combination of the actions performed by the group members is in the plan. Because the plan only contains optimal group actions, this combination of actions is an optimal group action. Hence, the group members perform an optimal group action. Therefore, if each group member fulfills her member obligation specified by a closed plan, then the group itself fulfills its group obligation. A closed plan thus solves the group members’ coordination problem.

Our account of the relation between group obligations, member obligations, and individual obligations has a number of notable features. First, it shows that fulfilling an individual obligation is neither necessary nor sufficient for fulfilling a member obligation. Secondly, if each group member fulfills her member obligation specified by a *closed* plan, then the group itself fulfills its group obligation. Thirdly, if a group does *not* fulfill its group obligation even though its members adopted a plan to solve their coordination problem, then one of three things has happened: (1) the adopted plan was *not* closed, each group member fulfilled her member obligation specified by the plan, but the combination of the actions performed by the group members was not an optimal group action; (2) the adopted plan was *not* closed, there was at least one group member who did not fulfill her member obligation specified by the plan, and the combination of the actions performed by the group members was not an optimal group action; or (3) the adopted plan *was* closed, there was at least one group member who did not fulfill her member obligation specified by the plan, and the combination of the actions performed by the group members was not an optimal group action. The third possibility supports the counterfactual ‘If each group member had fulfilled her member obligation specified by the plan, then the group itself would have fulfilled its group obligation’, while the second possibility does not. We submit that our account of member obligations helps us to better understand reductionism about collective moral responsibility.

## Reductionism revisited

Our analysis does not support logical reductionism about group obligations and individual obligations. Recall that logical reductionism concerns whether statements about collectives are logically equivalent to conjunctions of statements about individuals. As we showed in Sect. 3.3, group obligations do not imply (and nor are they implied by) individual obligations. Nonetheless, it might seem that our analysis supports logical reductionism about group obligations and *member* obligations: given a closed plan consisting of one or more optimal group actions, it holds that if each group member fulfills her member obligation specified by the plan, then the group itself fulfills its group obligation. Therefore, given that the closedness condition is met, the fulfillment of each member obligation implies the fulfillment of a group obligation. Is this a case of logical reductionism?

First, to meet the demands of logical reductionism, the converse conditional must also be valid. Indeed, Jackson argues that the conditional ‘If a group act is right, *and it is in fact performed*, then each individual constituent act is right’ is valid (Jackson 1987, p. 107). In the present setting, it is not. Given a closed plan consisting of one or more optimal group actions, it might be that the group fulfills its group obligation even though none of the group members fulfills her member obligation specified by the plan. The model of Fig. 2 provides a counterexample. Consider the plan $\{(a^{}_i,a^{}_j)\}$. Because it consists of a single optimal group action, it is closed. Suppose that agent *i* performs action $a^{\prime}_i$ and that agent *j* performs action $a^{\prime}_j$. Then the group performs the optimal group action $(a^{\prime}_i,a^{\prime}_j)$ even though neither agent *i* nor agent *j* fulfills her member obligation specified by the plan.

Secondly, the conditional ‘If each group member fulfills her member obligation specified by the plan, then the group itself fulfills its group obligation’ only holds *under the condition* that the plan is closed. If the plan is *not* closed, the conditional might be false: given a plan that is *not* closed, it might be that each group member fulfills her member obligation specified by the plan even though the group itself does not fulfill its group obligation. To see this, let us return to Fig. 2, where Angela and Bob will save a drowning person if and only if either (1) they both swim or (2) they both use the motorized dinghy. Given that Angela and Bob adopt the (bad!) group plan to save the drowning person either by swimming or by using the boat (that is, they adopt the group plan $\{(a^{}_i,a^{}_j),(a^{\prime}_i,a^{\prime}_j)\}$), it holds that if Angela were to swim ($a^{}_i$) and Bob were to use the boat ($a^{\prime}_j$), then both Angela and Bob would fulfill their member obligation specified by the plan, but the composition $(a^{}_i,a^{\prime}_j)$ of their individual actions would not amount to an optimal group action, and hence Angela and Bob would not fulfill their collective obligation.

Thirdly, an individualist might point to the fact that member obligations are obligations that individuals have and that this is all we need to vindicate logical reductionism. This response is wide of the mark, because a member obligation is of necessity a member obligation *specified by a plan consisting of one or more optimal group actions*. This means that a member obligation essentially includes a reference to a group. Group obligations are conceptually prior to member obligations, since the group members determine their member obligations by reasoning from the group obligation.^27^ As mentioned before, this kind of reasoning is called ‘we-reasoning’ (Tuomela 2013). Kutz acknowledges the role of such reasoning in determining member obligations when he writes that ‘[j]ointly acting agents must reason backwards from the nature of the group act to an understanding of what each should do if the group act is to be achieved’ (Kutz 2000a, pp. 83–84). It is hard to see how this position squares with his individualism.

Fourthly, an individualist might admit that member obligations include a reference to a group, but try to avoid the collectivist consequences of this admission by claiming that this reference is inessential, because member obligations are in fact individual obligations and individual obligations do not include any reference to a group. This response is inadequate for two reasons. First, the proposed identification of member obligations and individual obligations is not enough to meet the demands of individualism, because we showed in Sect. 3.3 that group obligations do not imply (and nor are they implied by) individual obligations. Secondly, we argued in Sect. 4 that fulfilling an individual obligation is neither necessary nor sufficient for fulfilling a member obligation specified by a plan: member obligations are not individual obligations.^28^

We conclude from these four considerations that our analysis does not support logical reductionism about group obligations and member obligations. Because logical reductionism about forward-looking and backward-looking collective moral responsibility is not to be had without logical reductionism about group obligations and member obligations, our analysis also fails to support logical reductionism about collective moral responsibility. All in all, our analysis vindicates collectivism about collective obligations and about collective moral responsibility.

## Figures and Tables

**Fig. 4:**
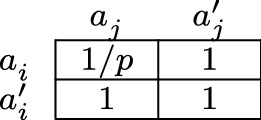
[*i*, *j*]*p*, but not [*i*]*p*

**Fig. 5:**
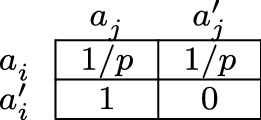
(*i*)*p*, but not (*i*, *j*)*p*

**Fig. 6:**
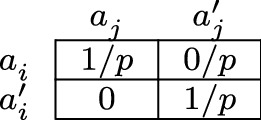
(*i*, *j*)*p*, but not (*i*)*p*

**Fig. 7:**
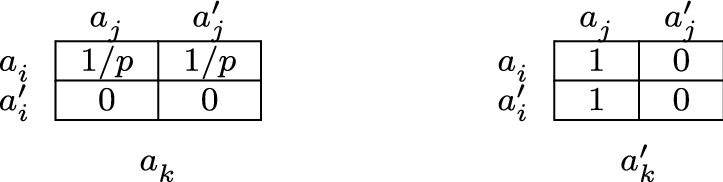
(*i*, *k*)*p* and $$(j,k)\lnot p$$

## Appendix

### **Lemma 1**

(Horty 2001, p. 74) *Let*$M=\langle N, (A^{}_i), d, v\rangle$*be a deontic game model*. *Let*${\mathcal {G}}\subseteq N$*be a group of agents*. *Then*

$$\begin{aligned} \textit{Optimal}({\mathcal {G}})\ne \emptyset . \end{aligned}$$

### *Proof*

Assume that $\textit{Optimal}({\mathcal {G}})=\emptyset$. By definition of *M*, it holds that $A^{}_{\mathcal {G}}$ is finite and non-empty. Then for all $a^{\prime}_{\mathcal {G}}\in A^{}_{\mathcal {G}}$ there is an $a^{}_{\mathcal {G}}\in A^{}_{\mathcal {G}}$ such that $a^{}_{\mathcal {G}}\succ a^{\prime}_{\mathcal {G}}$. Because $A^{}_{\mathcal {G}}$ is finite, there must be a cycle $a^n_{\mathcal {G}}\succ a^{n-1}_{\mathcal {G}}\succ \cdots \succ a^2_{\mathcal {G}}\succ a^1_{\mathcal {G}}=a^n_{\mathcal {G}}$ with all members $a^i_{\mathcal {G}}$ in $A^{}_{\mathcal {G}}$. Because $\succ$ is transitive, it holds that $a^n_{\mathcal {G}}\succ a^n_{\mathcal {G}}$, that is, $a^n_{\mathcal {G}}\succeq a^n_{\mathcal {G}}$ and $a^n_{\mathcal {G}}\not \succeq a^n_{\mathcal {G}}$. Contradiction. Therefore, $\textit{Optimal}({\mathcal {G}})\ne \emptyset$. $\square$

### **Lemma 2**

(Kooi and Tamminga 2008, p. 9) *Let*$M=\langle N, (A^{}_i), d, v\rangle$*be a deontic game model*. *Let*${\mathcal {F}}\subseteq {\mathcal {G}}\subseteq N$. *Let*$a^{}_{\mathcal {F}},a^{\prime}_{\mathcal {F}}\in A^{}_{\mathcal {F}}$*and let*$a^{\prime\prime}_{{\mathcal {G}}-{\mathcal {F}}}\in A^{}_{{\mathcal {G}}-{\mathcal {F}}}$. *Then*

$$\begin{aligned} If\,~a^{}_{\mathcal {F}}\succeq a^{\prime}_{\mathcal {F}}, then \, (a^{}_{\mathcal {F}},a^{\prime\prime}_{{\mathcal {G}}-{\mathcal {F}}})\succeq (a^{\prime}_{\mathcal {F}},a^{\prime\prime}_{{\mathcal {G}}-{\mathcal {F}}}). \end{aligned}$$

### *Proof*

Assume that $a^{}_{\mathcal {F}}\succeq a^{\prime}_{\mathcal {F}}$. Let $a^{\prime\prime}_{{\mathcal {G}}-{\mathcal {F}}}\in A^{}_{{\mathcal {G}}-{\mathcal {F}}}$. Suppose that $a^{\prime\prime\prime}_{-{\mathcal {G}}}\in A^{}_{-{\mathcal {G}}}$. Then $(a^{\prime\prime}_{{\mathcal {G}}-{\mathcal {F}}},a^{\prime\prime\prime}_{-{\mathcal {G}}})\in A^{}_{-{\mathcal {F}}}$. Hence, by our assumption it must be that $d(a^{}_{\mathcal {F}},a^{\prime\prime}_{{\mathcal {G}}-{\mathcal {F}}},a^{\prime\prime\prime}_{-{\mathcal {G}}})\ge d(a^{\prime}_{\mathcal {F}},a^{\prime\prime}_{{\mathcal {G}}-{\mathcal {F}}},a^{\prime\prime\prime}_{-{\mathcal {G}}})$. Therefore, for all $a^{\prime\prime\prime}_{-{\mathcal {G}}}\in A^{}_{-{\mathcal {G}}}$ it holds that $d(a^{}_{\mathcal {F}},a^{\prime\prime}_{{\mathcal {G}}-{\mathcal {F}}},a^{\prime\prime\prime}_{-{\mathcal {G}}})\ge d(a^{\prime}_{\mathcal {F}},a^{\prime\prime}_{{\mathcal {G}}-{\mathcal {F}}},a^{\prime\prime\prime}_{-{\mathcal {G}}})$. Hence, $(a^{}_{\mathcal {F}},a^{\prime\prime}_{{\mathcal {G}}-{\mathcal {F}}})\succeq (a^{\prime}_{\mathcal {F}},a^{\prime\prime}_{{\mathcal {G}}-{\mathcal {F}}})$. $\square$

### **Theorem 1**

*Let*$\phi ,\psi \in {\mathfrak {L}}$*and*${\mathcal {G}}\subseteq N$.* Then*

(i) $$\models ({\mathcal {G}})\phi \rightarrow \Diamond [{\mathcal {G}}]\phi $$
(ii) $$\models [{\mathcal {G}}](\phi \wedge \psi )\leftrightarrow ([{\mathcal {G}}]\phi \wedge [{\mathcal {G}}]\psi )$$
(iii) $$\models ({\mathcal {G}})(\phi \wedge \psi )\leftrightarrow (({\mathcal {G}})\phi \wedge ({\mathcal {G}})\psi )$$.

### *Proof*

(i) Assume that $M,a\models ({\mathcal {G}})\phi$. Then for all $a^{\prime}\in A$ with $a^{\prime}_{\mathcal {G}}\in \textit{Optimal}({\mathcal {G}})$ it holds that $M,a^{\prime}\models \phi$. Because $\textit{Optimal}({\mathcal {G}})\ne \emptyset$, there is an $a^{\prime}_{\mathcal {G}}\in \textit{Optimal}({\mathcal {G}})$. Suppose that $a^{\prime\prime}\in A$ is such that $a^{\prime\prime}_{\mathcal {G}}=a^{\prime}_{\mathcal {G}}$. Then it must be that $M,a^{\prime\prime}\models \phi$. Hence, for all $a^{\prime\prime}\in A$ with $a^{\prime\prime}_{\mathcal {G}}=a^{\prime}_{\mathcal {G}}$ it holds that $M,a^{\prime\prime}\models \phi$. Then $M,a^{\prime}\models [{\mathcal {G}}]\phi$. Hence, there is an $a^{\prime}\in A$ such that $M,a^{\prime}\models [{\mathcal {G}}]\phi$. Therefore, $M,a\models \Diamond [{\mathcal {G}}]\phi$.

(ii) ($\Rightarrow$) Assume that $M,a\models [{\mathcal {G}}](\phi \wedge \psi )$. Then for all $a^{\prime}\in A$ with $a^{\prime}_{\mathcal {G}}=a^{}_{\mathcal {G}}$ it holds that $M,a^{\prime}\models \phi \wedge \psi$. Then for all $a^{\prime}\in A$ with $a^{\prime}_{\mathcal {G}}=a^{}_{\mathcal {G}}$ it holds that $M,a^{\prime}\models \phi$ and for all $a^{\prime}\in A$ with $a^{\prime}_{\mathcal {G}}=a^{}_{\mathcal {G}}$ it holds that $M,a^{\prime}\models \psi$. Hence, it must be that $M,a\models [{\mathcal {G}}]\phi$ and $M,a\models [{\mathcal {G}}]\psi$. Therefore, $M,a\models [{\mathcal {G}}]\phi \wedge [{\mathcal {G}}]\psi$.

($\Leftarrow$) Assume that $M,a\models [{\mathcal {G}}]\phi \wedge [{\mathcal {G}}]\psi$. Then $M,a\models [{\mathcal {G}}]\phi$ and $M,a\models [{\mathcal {G}}]\psi$. Then for all $a^{\prime}\in A$ with $a^{\prime}_{\mathcal {G}}=a^{}_{\mathcal {G}}$ it holds that $M,a^{\prime}\models \phi$ and for all $a^{\prime}\in A$ with $a^{\prime}_{\mathcal {G}}=a^{}_{\mathcal {G}}$ it holds that $M,a^{\prime}\models \psi$. Then for all $a^{\prime}\in A$ with $a^{\prime}_{\mathcal {G}}=a^{}_{\mathcal {G}}$ it holds that $M,a^{\prime}\models \phi \wedge \psi$. Therefore, $M,a\models [{\mathcal {G}}](\phi \wedge \psi )$.

(iii) ($\Rightarrow$) Assume that $M,a\models ({\mathcal {G}})(\phi \wedge \psi )$. Then for all $a^{\prime}\in A$ with $a^{\prime}_{\mathcal {G}}\in \textit{Optimal}({\mathcal {G}})$ it holds that $M,a^{\prime}\models \phi \wedge \psi$. Then for all $a^{\prime}\in A$ with $a^{\prime}_{\mathcal {G}}\in \textit{Optimal}({\mathcal {G}})$ it holds that $M,a^{\prime}\models \phi$ and for all $a^{\prime}\in A$ with $a^{\prime}_{\mathcal {G}}\in \textit{Optimal}({\mathcal {G}})$ it holds that $M,a^{\prime}\models \psi$. Hence, it must be that $M,a\models ({\mathcal {G}})\phi$ and $M,a\models ({\mathcal {G}})\psi$. Therefore, $M,a\models ({\mathcal {G}})\phi \wedge ({\mathcal {G}})\psi$.

($\Leftarrow$) Assume that $M,a\models ({\mathcal {G}})\phi \wedge ({\mathcal {G}})\psi$. Then $M,a\models ({\mathcal {G}})\phi$ and $M,a\models ({\mathcal {G}})\psi$. Then for all $a^{\prime}\in A$ with $a^{\prime}_{\mathcal {G}}\in \textit{Optimal}({\mathcal {G}})$ it holds that $M,a^{\prime}\models \phi$ and for all $a^{\prime}\in A$ with $a^{\prime}_{\mathcal {G}}\in \textit{Optimal}({\mathcal {G}})$ it holds that $M,a^{\prime}\models \psi$. Then for all $a^{\prime}\in A$ with $a^{\prime}_{\mathcal {G}}\in \textit{Optimal}({\mathcal {G}})$ it holds that $M,a^{\prime}\models \phi \wedge \psi$. Therefore, $M,a\models ({\mathcal {G}})(\phi \wedge \psi )$. $\square$

### **Theorem 2**

*Let*$$\phi \in {\mathfrak {L}}$$. *Then*

(i) $$\models [{\mathcal {F}}]\phi \rightarrow [{\mathcal {G}}]\phi $$ for all $${\mathcal {F}}\subseteq {\mathcal {G}}\subseteq N$$
(ii) $\not \models [{\mathcal {G}}]\phi \rightarrow [{\mathcal {F}}]\phi$ for some ${\mathcal {F}}\subseteq {\mathcal {G}}\subseteq N$.

### *Proof*

(i) Assume that $M,a\models [{\mathcal {F}}]\phi$. Then for all $a^{\prime}\in A$ with $a^{\prime}_{\mathcal {F}}=a^{}_{\mathcal {F}}$ it holds that $M,a^{\prime}\models \phi$. Suppose that $a^{\prime\prime}\in A$ is such that $a^{\prime\prime}_{\mathcal {G}}=a^{}_{\mathcal {G}}$. Note that $a^{}_{\mathcal {G}}$ can be written as $(a^{}_{\mathcal {F}},a^{}_{{\mathcal {G}}-{\mathcal {F}}})$. Then it must be that $a^{\prime\prime}=(a^{}_{\mathcal {F}},a^{}_{{\mathcal {G}}-{\mathcal {F}}},a^{\prime\prime}_{-{\mathcal {G}}})$ and $a^{\prime\prime}_{\mathcal {F}}=a^{}_{\mathcal {F}}$. Then it must be that $M,a^{\prime\prime}\models \phi$. Hence, for all $a^{\prime\prime}\in A$ with $a^{\prime\prime}_{\mathcal {G}}=a^{}_{\mathcal {G}}$ it holds that $M,a^{\prime\prime}\models \phi$. Therefore, $M,a\models [{\mathcal {G}}]\phi$.

(ii) We define a model $$M=\langle N, (A^{}_i), d, v\rangle $$. Let $$N=\{i,j\}$$ be its set of agents. Let $$A^{}_i=\{a^{}_i,a^{\prime}_i\}$$ be the set of actions available to agent *i* and let $$A^{}_j=\{a^{}_j,a^{\prime}_j\}$$ be the set of actions available to agent *j*. Let the deontic ideality function *d* be defined as follows: $$d(a^{}_i,a^{}_j)$$$$=$$$$d(a^{}_i,a^{\prime}_j)$$$$=$$$$d(a^{\prime}_i,a^{}_j)$$$$=$$$$d(a^{\prime}_i,a^{\prime}_j)$$$$=$$ 1. Let the valuation function *v* be defined as follows: $$v(p)=\{(a^{}_i,a^{}_j)\}$$. See Fig. 4. Then it is easy to see that $$M,(a^{}_i,a^{}_j)\models [i,j]p$$ and $$M,(a^{}_i,a^{}_j)\not \models [i]p$$. $$\square $$

### **Theorem 3**

*Let*$$\phi \in {\mathfrak {L}}$$. *Then*

(i) $$\not \models ({\mathcal {F}})\phi \rightarrow ({\mathcal {G}})\phi $$ for some $${\mathcal {F}}\subseteq {\mathcal {G}}\subseteq N$$
(ii) $\not \models ({\mathcal {G}})\phi \rightarrow ({\mathcal {F}})\phi$ for some ${\mathcal {F}}\subseteq {\mathcal {G}}\subseteq N$.

### *Proof*

(i) Following Horty (2001, p. 126), we define a model $$M=\langle N, (A^{}_i), d, v\rangle $$. Let $$N=\{i,j\}$$ be its set of agents. Let $$A^{}_i=\{a^{}_i,a^{\prime}_i\}$$ be the set of actions available to agent *i* and let $$A^{}_j=\{a^{}_j,a^{\prime}_j\}$$ be the set of actions available to agent *j*. Let the deontic ideality function *d* be defined as follows: $$d(a^{}_i,a^{}_j)$$$$=$$$$d(a^{}_i,a^{\prime}_j)$$$$=$$$$d(a^{\prime}_i,a^{}_j)$$$$=$$ 1 and $$d(a^{\prime}_i,a^{\prime}_j)=0$$. Then $$\textit{Optimal}(i)=\{a^{}_i\}$$ and $$\textit{Optimal}(i,j)=\{(a^{}_i,a^{}_j),(a^{}_i,a^{\prime}_j),(a^{\prime}_i,a^{}_j)\}$$. Let the valuation function *v* be defined as follows: $$v(p)=\{(a^{}_i,a^{}_j),(a^{}_i,a^{\prime}_j)\}$$. See Fig. 5. Then it is easy to see that for all action profiles *a* it holds that $$M,a\models (i)p$$ and $$M,a\not \models (i,j)p$$.

(ii) Following Horty (2001, p. 125), we define a model $$M=\langle N, (A^{}_i), d, v\rangle $$. Let $$N=\{i,j\}$$ be its set of agents. Let $$A^{}_i=\{a^{}_i,a^{\prime}_i\}$$ be the set of actions available to agent *i* and let $$A^{}_j=\{a^{}_j,a^{\prime}_j\}$$ be the set of actions available to agent *j*. Let the deontic ideality function *d* be defined as follows: $$d(a^{}_i,a^{}_j)$$$$=$$$$d(a^{\prime}_i,a^{\prime}_j)$$$$=$$ 1 and $$d(a^{}_i,a^{\prime}_j)$$$$=$$$$d(a^{\prime}_i,a^{}_j)$$$$=$$ 0. Then $$\textit{Optimal}(i)=\{a^{}_i,a^{\prime}_i\}$$ and $$\textit{Optimal}(i,j)=\{(a^{}_i,a^{}_j),(a^{\prime}_i,a^{\prime}_j)\}$$. Let the valuation function *v* be defined as follows: $$v(p)=\{(a^{}_i,a^{}_j),(a^{}_i,a^{\prime}_j),(a^{\prime}_i,a^{\prime}_j)\}$$. See Fig. 6. Then it is easy to see that for all action profiles *a* it holds that $$M,a\models (i,j)p$$ and $$M,a\not \models (i)p$$. $$\square $$

### **Theorem 4**

*Let*$\phi \in {\mathfrak {L}}$*and*${\mathcal {F}}\subseteq {\mathcal {G}}\subseteq N$. *Then*

(i) $$\models ({\mathcal {F}})\phi \rightarrow \lnot ({\mathcal {G}})\lnot \phi $$
(ii) $$\models ({\mathcal {G}})\phi \rightarrow \lnot ({\mathcal {F}})\lnot \phi $$.

### *Proof*

(i) Assume that $M,a\models ({\mathcal {F}})\phi$. Then for all $a^{\prime}\in A$ with $a^{\prime}_{\mathcal {F}}\in \textit{Optimal}({\mathcal {F}})$ it holds that $M,a^{\prime}\models \phi$. Suppose that $M,a\models ({\mathcal {G}})\lnot \phi$. Then for all $a^{\prime}\in A$ with $a^{\prime}_{\mathcal {G}}\in \textit{Optimal}({\mathcal {G}})$ it holds that $M,a^{\prime}\models \lnot \phi$. Note that $\textit{Optimal}({\mathcal {G}})\ne \emptyset$. Let $a^{\prime}\in A$ be such that $a^{\prime}_{\mathcal {G}}\in \textit{Optimal}({\mathcal {G}})$. Note that $a^{\prime}_{\mathcal {G}}=(a^{\prime}_{\mathcal {F}},a^{\prime}_{{\mathcal {G}}-{\mathcal {F}}})$ for an $a^{\prime}_{\mathcal {F}}\in A^{}_{\mathcal {F}}$ and an $a^{\prime}_{{\mathcal {G}}-{\mathcal {F}}}\in A^{}_{{\mathcal {G}}-{\mathcal {F}}}$. Suppose that $a^{\prime}_{\mathcal {F}}\in \textit{Optimal}({\mathcal {F}})$. Because $a^{\prime}_{\mathcal {F}}\in \textit{Optimal}({\mathcal {F}})$, it holds that $M,a^{\prime}\models \phi$. Because $(a^{\prime}_{\mathcal {F}},a^{\prime}_{{\mathcal {G}}-{\mathcal {F}}})=a^{\prime}_{\mathcal {G}}\in \textit{Optimal}({\mathcal {G}})$, it holds that $M,a^{\prime}\models \lnot \phi$. Contradiction. Hence, $a^{\prime}_{\mathcal {F}}\not \in \textit{Optimal}({\mathcal {F}})$. Then there must be an $a^{\prime\prime}\in A$ with $a^{\prime\prime}_{\mathcal {F}}\in \textit{Optimal}({\mathcal {F}})$ such that $a^{\prime\prime}_{\mathcal {F}}\succ a^{\prime}_{\mathcal {F}}$. Hence, $a^{\prime\prime}_{\mathcal {F}}\succeq a^{\prime}_{\mathcal {F}}$. By Lemma 2, it must be that $(a^{\prime\prime}_{\mathcal {F}},a^{\prime}_{{\mathcal {G}}-{\mathcal {F}}})\succeq (a^{\prime}_{\mathcal {F}},a^{\prime}_{{\mathcal {G}}-{\mathcal {F}}})=a^{\prime}_{\mathcal {G}}$. Note that for all $a^{\prime\prime\prime}\in A$ with $a^{\prime\prime\prime}_{\mathcal {F}}=a^{\prime\prime}_{\mathcal {F}}$ it holds that $M,a^{\prime\prime\prime}\models \phi$ and that $(a^{\prime\prime}_{\mathcal {F}},a^{\prime}_{{\mathcal {G}}-{\mathcal {F}}})\in A^{}_{\mathcal {G}}$. Hence, $(a^{\prime\prime}_{\mathcal {F}},a^{\prime}_{{\mathcal {G}}-{\mathcal {F}}})\not \in \textit{Optimal}({\mathcal {G}})$. Then there must be an $a^*\in A$ with $a^*_{\mathcal {G}}\in \textit{Optimal}({\mathcal {G}})$ such that $a^*_{\mathcal {G}}\succ (a^{\prime\prime}_{\mathcal {F}},a^{\prime}_{{\mathcal {G}}-{\mathcal {F}}})$. Because $\succ$ is transitive, it must be that $a^*_{\mathcal {G}}\succ a^{\prime}_{\mathcal {G}}$. Hence, $a^{\prime}_{\mathcal {G}}\not \in \textit{Optimal}({\mathcal {G}})$. Contradiction. Therefore, $M,a\not \models ({\mathcal {G}})\lnot \phi$. Hence, $M,a\models \lnot ({\mathcal {G}})\lnot \phi$.

(ii) Analogous to (i). $\square$

### **Theorem 5**

*Let*$$\phi \in {\mathfrak {L}}$$. *Then*

(i) $\models ({\mathcal {F}}^{}_1)\phi \rightarrow \lnot ({\mathcal {F}}^{}_2)\lnot \phi$    for all ${\mathcal {F}}^{}_1,{\mathcal {F}}^{}_2\subseteq N$ such that ${\mathcal {F}}^{}_1\cap {\mathcal {F}}^{}_2=\emptyset$
(ii) $\not \models ({\mathcal {F}}^{}_1)\phi \rightarrow \lnot ({\mathcal {F}}^{}_2)\lnot \phi$    for some ${\mathcal {F}}^{}_1,{\mathcal {F}}^{}_2\subseteq N$
(iii) $\models (({\mathcal {F}}^{}_1)\phi \wedge ({\mathcal {F}}^{}_2)\lnot \phi )\rightarrow (\lnot ({\mathcal {G}})\phi \wedge \lnot ({\mathcal {G}})\lnot \phi )$    for all ${\mathcal {F}}^{}_1,{\mathcal {F}}^{}_2\subseteq {\mathcal {G}}\subseteq N$
(iv) $\models (({\mathcal {G}})\phi \vee ({\mathcal {G}})\lnot \phi )\rightarrow \lnot (({\mathcal {F}}^{}_1)\phi \wedge ({\mathcal {F}}^{}_2)\lnot \phi )$      for all ${\mathcal {F}}^{}_1,{\mathcal {F}}^{}_2\subseteq {\mathcal {G}}\subseteq N$.

### *Proof*

(i) Following Horty (2001, p. 79), assume that $M,a\models ({\mathcal {F}}^{}_1)\phi$. Then for all $a^{\prime}\in A$ with $a^{\prime}_{{\mathcal {F}}^{}_1}\in \textit{Optimal}({\mathcal {F}}^{}_1)$ it holds that $M,a^{\prime}\models \phi$. Because $\textit{Optimal}({\mathcal {F}}^{}_1)\ne \emptyset$, there is an $a^{\prime}\in A$ with $a^{\prime}_{{\mathcal {F}}^{}_1}\in \textit{Optimal}({\mathcal {F}}^{}_1)$. Suppose that $M,a\models ({\mathcal {F}}^{}_2)\lnot \phi$. Then for all $a^{\prime\prime}\in A$ with $a^{\prime\prime}_{{\mathcal {F}}_2}\in \textit{Optimal}({\mathcal {F}}^{}_2)$ it holds that $M,a^{\prime\prime}\models \lnot \phi$. Because $\textit{Optimal}({\mathcal {F}}^{}_2)\ne \emptyset$, there is an $a^{\prime\prime}\in A$ with $a^{\prime\prime}_{{\mathcal {F}}^{}_2}\in \textit{Optimal}({\mathcal {F}}^{}_2)$. Because ${\mathcal {F}}^{}_1\cap {\mathcal {F}}^{}_2 =\,\emptyset$, it holds that $(a^{\prime}_{{\mathcal {F}}_1},a^{\prime\prime}_{{\mathcal {F}}_2})\in A^{}_{{\mathcal {F}}_1\cup {\mathcal {F}}_2}$. Let $a^*=(a^{\prime}_{{\mathcal {F}}_1},a^{\prime\prime}_{{\mathcal {F}}_2},a^{\prime\prime\prime}_{-({\mathcal {F}}_1\cup {\mathcal {F}}_2)})$ for some $a^{\prime\prime\prime}\in A$ with $a^{\prime\prime\prime}_{-(F_1\cup {\mathcal {F}}_2)}\in A^{}_{-(F_1\cup {\mathcal {F}}_2)}$. Then $M,a^*\models \phi$ and $M,a^*\models \lnot \phi$. Contradiction. Therefore, $M,a\models \lnot ({\mathcal {F}}_2)\lnot \phi$.

(ii) Following Kooi and Tamminga (2008, p. 16), we define a model $$M=\langle N, (A^{}_i), d, v\rangle $$. Let $$N=\{i,j,k\}$$ be its set of agents. Let $$A^{}_i=\{a^{}_i,a^{\prime}_i\}$$ be the set of actions available to agent *i*, let $$A^{}_j=\{a^{}_j,a^{\prime}_j\}$$ be the set of actions available to agent *j*, and let $$A^{}_k=\{a^{}_k,a^{\prime}_k\}$$ be the set of actions available to agent *k*. Let the deontic ideality function *d* be defined as follows: $$d(a^{}_i,a^{}_j,a^{}_k)$$$$=$$$$d(a^{}_i,a^{}_j,a^{\prime}_k)$$$$=$$$$d(a^{}_i,a^{\prime}_j,a^{}_k)$$$$=$$$$d(a^{\prime}_i,a^{}_j,a^{\prime}_k)$$$$=$$ 1 and $$d(a^{}_i,a^{\prime}_j,a^{\prime}_k)$$$$=$$$$d(a^{\prime}_i,a^{}_j,a^{}_k)$$$$=$$$$d(a^{\prime}_i,a^{\prime}_j,a^{}_k)$$$$=$$$$d(a^{\prime}_i,a^{\prime}_j,a^{\prime}_k)$$$$=$$ 0. Let the valuation function *v* be defined as follows: $$v(p)=\{(a^{}_i,a^{}_j,a^{}_k),(a^{}_i,a^{\prime}_j,a^{}_k)\}$$. See Fig. 7. Then it is easy to see that for all action profiles *a* it holds that $$M,a\models (i,k)p$$ and $$M,a\models (j,k)\lnot p$$.

(iii) Use Theorem 4 and contraposition.

(iv) Use (iii) and contraposition. $\square$

## Footnotes

1 Copp (2006), List and Pettit (2011), and Hindriks (2013) argue against ontological reductionism about structured groups.

2 Not fulfilling an obligation, however, is not a sufficient condition for being individually blameworthy: the agent might have a justification or an excuse for not doing her duty. Furthermore, whereas blame requires not fulfilling an obligation, supererogatory actions make it clear that praise does not require fulfilling an obligation.

3 Feinberg argues that such collective responsibility cannot be reduced to individual responsibility, whereas Held defends reductionism about collective responsibility.

4 Held argues that ‘from the judgment “Collectivity *C* ought (ought not) to have done *A*”, judgments of the form “Member *M* of *C* ought (ought not) to have done *A*” cannot be derived’ and that ‘judgments about the moral responsibility of its members are not logically derivable from judgments about the moral responsibility of a collectivity’ (Held 1970, pp. 474–475). Compare Gilbert: ‘What does the blameworthiness of the collective’s act imply about the personal blameworthiness of any one member of that collective? From a logical point of view, the short answer is: *nothing*. Everything depends on the details of a given member’s particular situation’ (Gilbert 2006, p. 109). Likewise, Isaacs argues that ‘claims about collective moral responsibility neither entail nor are derivable from claims about individual moral responsibility’ (Isaacs 2011, p. 24).

5 Our deontic logic of collective action stands in the tradition of the *stit* (‘sees to it that’) logics of agency developed by Kanger (1957), Pörn (1970), Kanger (1972), Hansson (1986), von Kutschera (1986), Horty and Belnap (1995), Belnap et al. (2001), Horty (2001), Kooi and Tamminga (2008), Carmo (2010), and Tamminga (2013).

6 Collective intentions play a central role in the analysis of joint actions. See, for instance, Tuomela and Miller (1988), Gilbert (1990), Searle (1990), and Bratman (1992). For analyses of collective action that lack collective intentions, see Jackson (1987), Kutz (2000b), and Chant (2007).

7 For a formal analysis of the concept of individual ability, see Horty (2001, pp. 19–29).

8 We require finiteness for expository reasons only. If we were to admit infinite sets of individual actions, we would have to generalize the truth-condition for deontic statements accordingly—see footnote 12.

9 We adopt the notational conventions of Osborne and Rubinstein (1994, Section 1.7).

10 The deontic ideality function represents a given moral code. Our binary ordering of the action profiles in terms of deontic ideality can also be seen to reflect a simple preference ordering of agents who classify action profiles unanimously as ‘good’ or ‘bad’. Deontic game models thus resemble Schelling’s (1960, p. 84) *pure-collaboration games* and Bacharach’s (2006, p. 122) *coordination contexts*.

11 See Hansson (1986), Tuomela (1989), Belnap et al. (2001, Ch. 10), Horty (2001, pp. 30–33), and Carmo (2010).

12 If we were to admit infinite sets of individual actions (and hence admit infinite sets of action profiles), then the truth-condition for deontic statements would be as follows: $M,a\models ({\mathcal {G}})\phi$ if and only if for all $a^{\prime}\in A$ with $M,a^{\prime}\not \models \phi$ there is an $a^{\prime\prime}\in A$ with $M,a^{\prime\prime}\models \phi$ such that (i) $a^{\prime\prime}_{\mathcal {G}}\succ a^{\prime}_{\mathcal {G}}$ and (ii) for all $a^{\prime\prime\prime}\in A$ with $a^{\prime\prime\prime}_{\mathcal {G}}\succeq a^{\prime\prime}_{\mathcal {G}}$ it holds that $M,a^{\prime\prime\prime}\models \phi$. Compare Horty (2001, p. 77). This generalized truth-condition preserves the theorems below that, at first sight, seem to rely on the finiteness condition—Theorems 1(i), 4(i), 4(ii) and 5(i).

13 That is, every modality $\star$ such that $\star \in \{\Box ,[{\mathcal {G}}],({\mathcal {G}})\}$ validates the principle of necessitation: ‘if $\models \phi$, then $\models \star \phi$’, and the principle of distribution over implication: ‘$\models \star (\phi \rightarrow \psi )\rightarrow (\star \phi \rightarrow \star \psi )$’.

14 We could introduce two additional non-normal operators, ‘Group ${\mathcal {G}}$ of agents deliberately sees to it that $\phi$’ (formalized as $[\![{\mathcal {G}}]\!]\phi$) and ‘Group ${\mathcal {G}}$ of agents deliberately ought to see to it that $\phi$’ (formalized as $(\!({\mathcal {G}})\!)\phi$), and stipulate the following truth-conditions for them: $M,a\models [\![{\mathcal {G}}]\!]\phi$ if and only if $M,a\models [{\mathcal {G}}]\phi \wedge \lnot \Box \phi$, and $M,a\models (\!({\mathcal {G}})\!)\phi$ if and only if $M,a\models ({\mathcal {G}})\phi \wedge \lnot \Box \phi$. The validities for these new operators are the same as the ones listed below, with two exceptions: in Theorems 1(ii) and 1(iii), the condition $\lnot \Box \phi \wedge \lnot \Box \psi$ must be added to the left-hand side of the bi-conditional. Horty and Belnap (1995) study the single-agent version of the non-normal agentive modality $[\![{\mathcal {G}}]\!]$.

15 Ethical theorists debate whether the principle of ‘ought’ implies ‘can’ holds in every conceivable situation. Key references include Kekes (1984), Sinnott-Armstrong (1988), Howard-Snyder (2006), and Graham (2011).

16 This seems awkward as long as we do not make the following distinction: ‘[t]here is more than one thing that one might mean by $[{\mathcal {G}}]\phi$. First, one might mean that the bearers of ${\mathcal {G}}$, without any outside help, guarantee that $\phi$, on the basis of a prior simultaneous real choice by each of them. There is also a second, stronger, account. In this version the bearers of ${\mathcal {G}}$, without any outside help, *and with the essential input of each of them*, guarantee that $\phi$’ (Belnap et al. 2001, p. 282—notation adapted). We model the first of these two notions of group action.

17 Regan claims that ‘all subgroups of any group which produces best consequences produce best consequences also’ and, moreover, that this statement is equivalent to the statement that ‘there can be no conflict between the production of good consequences by large groups and by smaller included groups’ (Regan 1980, p. 108). Compare Regan (1980, pp. 54 and 56). In the present setting, Regan’s first statement is similar to $({\mathcal {G}})\phi \rightarrow ({\mathcal {F}})\phi$, which is invalid (Theorem 3(ii)). His second statement is similar to $({\mathcal {G}})\phi \rightarrow \lnot ({\mathcal {F}})\lnot \phi$, which is valid (Theorem 4(ii)). For a detailed comparison between Regan’s construction of obligations and one that is closely related to ours, see Horty (2001, Ch. 6).

18 See Collins (2013, p. 234) for a similar beach example.

19 If we read ‘direct wrongdoing’ as not fulfilling an individual obligation and ‘participatory wrongdoing’ as not fulfilling a member obligation, then we do something here that Kutz deems impossible: ‘[w]e will never find a single argument for distinguishing between participatory and direct wrongdoing, for the simple reason that there is no single framework for assessing accountability’ (Kutz 2000a, p. 148).

20 See Collins (2017) for an informal, but more comprehensive, analysis of ‘membership duties’.

21 Held argues that a random collection of agents might even have a duty to do so: ‘the random collection can be held morally responsible for failing to make a decision on which action to take’ (Held 1970, p. 479). See also Collins (2013) and Hindriks (2019).

22 In the theoretical economics literature, the preferred term is *team reasoning* (Bacharach 1999, 2006; Sugden 1993, 2000, 2003). Bacharach (2006, p. 121) writes: ‘[r]oughly, somebody “team-reasons” if she *works out the best feasible combination of actions for all the members of her team, then does her part in it*.’ Hakli et al. (2010) provide a detailed comparison of we-reasoning and team reasoning.

23 For clarity, we suppress the valuation function of the models.

24 Compare Regan: ‘[i]f the members of ${\mathcal {G}}$ all do their part in the best pattern of behaviour for the members of ${\mathcal {G}}$ given the behaviour of non-members, it is clear that the members of ${\mathcal {G}}$ produce the best consequences possible as a group’ (Regan 1980, p. 138—notation adapted).

25 This is a possible interpretation of the principle ‘If a group act is wrong, at least one of its constituent individual acts is wrong’ (Jackson 1987, p. 107). On the basis of an informal counterexample, Jackson argues that this principle is *invalid* (Jackson 1987, pp. 102–103). In our view, Jackson’s counterexample does *not* invalidate the principle, because it relies on different concepts of obligations—group obligations, member obligations, individual obligations, conditional individual obligations—that are not systematically distinguished.

26 Our concept of closedness goes back to Nash (1951, p. 290), where it is called *interchangeability*.

27 Copp, Isaacs, and Lawford-Smith defend closely related positions on the connection between group obligations and member obligations. Copp (2007, p. 374) writes: ‘if a collective has a duty, this is itself a moral consideration that gives at least some members a pro tanto duty to do their part in bringing it about that the collective does its duty’. Isaacs (2011, p. 10) maintains that ‘collective obligation lends shape and form to the obligations of individuals’. Compare Isaacs (2011, pp. 57–58). Lawford-Smith (2012, p. 463) argues that ‘[g]roups have an obligation, which divides into parts in its bearing on the parts of the group, the members’.

28 Our analysis ties in with Copp’s (2006, 2007) claim that individual obligations might conflict with and even outweigh member obligations. Copp argues that member obligations are *pro tanto* obligations rather than all-things-considered obligations. Accordingly, it might be that a group has an all-things-considered obligation and that the group members have *pro tanto* member obligations. Such a *pro tanto* member obligation might be outweighed by an all-things-considered individual obligation.
